# Surgery

**Published:** 1896-04

**Authors:** 


### SURGERY.

EDITED BY FLOYD W. MCRAE, M.D.

### THE INCREASE OF CANCER.

Mr. Roger Williams, F.R.C.S., in a recent paper read before the Lancashire and Cheshire Branch of the British Medical Association, calls attention to the continuously progressive increase in the cancer mortality. He shows that whereas in 1840 the cancer death-rate was 177 per 1,000,000 living, in 1893 it had risen to 711, and that the proportion of cancer deaths to total deaths had risen from 1 to 129 to 1 to 27 during the same period. He says that, if unchecked, cancer will erelong become one of the commonest diseases of modern communities. Even now cancer causes more than four times as many deaths as typhoid fever; but while medical officers of health devote so much attention in their reports to the latter disease—which is decreasing—they hardly ever allude to the former—and I believe they consider cancer outside their sphere. Mr. Williams calculates that there cannot be fewer than 42,000 persons now suffering from cancer in England and Wales; whereas in 1840 the number was only about 5,500. This increase he cannot account for by mere increase of population, as its increase has been proportionately much in excess of the population increase; nor can it, he thinks, be ascribed to improved diagnosis or other casual error.

Attention is directed to the remarkable decline in the death-rate from phthisis and other tubercular diseases that has coincided with the great increase in the cancer mortality. The latest ascertained cancer death-rate is the highest on record, while that from tubercle is the lowest. "I regard," he says, "this decline in the prevalence of tubercular disease as the outcome of improved hygienic conditions, due to augmented national prosperity, which, as I have elsewhere endeavored to show, by its action in another direction, is also responsible for the increased cancer mortality." Thus there appears to be some truth in the curious paradox that a high cancer mortality is an indication of good sanitary conditions.—*Quarterly Medical Journal.*

---

THE TREATMENT OF SPRAINED ANKLE BY ADHESIVE STRIPS.

This method of treating sprained ankle, which is said to have originated with Dr. Wm. R. Davis, U. S. A., is thus described by Dr. V. P. Gibney, who has done much to perfect it (*New York Medical Journal*): For a sprain above the external malleolus, a strip of rubber plaster about twelve inches in length is applied, beginning at the outer border of the foot, near the little toe, and extending to the inner side of the foot, above its middle, just above the plantar arch. The second strip is applied vertically, and passes from the junction of the middle, with the lower third of the leg down alongside of the Tendo Achillis, over the heel, and terminating at a point just above the internal malleolus; but posterior to this, other strips are applied in the same way, one overlapping the other about one-half, until the whole external malleolus and side of the foot up to the middle third of the leg is covered. It is well to reinforce just above the malleolus, by strips passing criss-cross, for additional support. The strips should well overlap each other, especially over the tendon Achillis and heel. The ankle should not be completely encircled, so as to avoid constriction. When the sprain involves the tarsal or metatarsal joint and the whole foot is edematous, the strips are applied as follows: The first one is placed on the inner side of the heel, passes back to the heel below the external malleolus, over the dorsum of the foot, and terminates u st under the ball of the great toe. The second is started under the external malleolus, passes over the back of the heel, over the foot, and terminates under the outer side of the foot, near the small toe. Sometimes extra strips are also applied up and down the tendon Achillis, the ends terminating on the sole of the foot. Over the ankle thus strapped a cheese cloth bandage is simply applied, up to the middle third of the leg.

---

### GASTRIC, INTESTINAL, AND RECTAL HEMORRHAGE.

In a paper (*Mathews Quarterly*) Dr. T. H. Manley, of New York, closes with the following summary:

First. Hemorrhage from the rectum may be symptomatic, of constitutional or organic disease, as plethora or hepatic congestion, etc.

Secondly. In consequence of a lesion of some part of the digestive tube, anywhere from the flexure to the cardiac end of the stomach, bleeding may escape, changed or unchanged, through the rectum.

Thirdly. The local lesions, in their order of frequency, as a source of hemorrhage, in the ileo-rectal outlet of the intestines are: (a) hemorrhoids, (b) simple or tubercular *ulceration*, (d) malignant disease.

*Treatment includes constitutional and local measures.*

Hemorrhage from simple, tubercular, cancerous, dysenteric, or typhoidal ulceration, in any part of the digestive tube, above the rectum, is quite beyond relief from direct surgical methods, and hence its treatment must, for the present at least, remain within the domain of medicine.

Surgical treatment of hemorrhage of the rectal-pouch and anus when non-malignant is generally *practicable*, safe, and permanent in results. In order, however, to be rendered effectual and definite thorough *dilatation* of the anus and eversion of the rectum are imperative in order that the bleeding points or source of hemorrhage may be brought under the immediate eye for direct and radical measures.

When bleeding succeeds from hemorrhoids for the first time, or when its quantity is small, moderate catharsis, with simple astringents in the form of suppositories, will favor its arrest, without recourse to radical or severe methods.

